# Electronic, magnetic, optical and thermoelectric properties of Ca_2_Cr_1−*x*_Ni_*x*_OsO_6_ double perovskites†

**DOI:** 10.1039/c9ra10775d

**Published:** 2020-04-23

**Authors:** Shalika R. Bhandari, D. K. Yadav, B. P. Belbase, M. Zeeshan, B. Sadhukhan, D. P. Rai, R. K. Thapa, G. C. Kaphle, Madhav Prasad Ghimire

**Affiliations:** Central Department of Physics, Tribhuvan University Kathmandu Nepal mpghimire@tucdp.edu.np ghimire.mpg@gmail.com; Institute for Theoretical Solid State Physics, IFW Dresden e. V. Dresden-01069 Germany; Condensed Matter Physics Research Center (CMPRC) Butwal Rupandehi Nepal; Department of Chemistry, Indian Institute of Technology Roorkee Roorkee 247667 Uttarakhand India; Physical Sciences Research Center (PSRC), Department of Physics, Pachhunga University College Aizawl Mizoram India

## Abstract

With the help of density functional theory calculations, we explored the recently synthesized double perovskite material Ca_2_CrOsO_6_ and found it to be a ferrimagnetic insulator with a band gap of ∼0.6 eV. Its effective magnetic moment is found to be ∼0.23 *μ*_B_ per unit cell. The proposed behavior arises from the cooperative effect of spin–orbit coupling and Coulomb correlation of Cr-3d and Os-5d electrons along with the crystal field. Within the ferrimagnetic configuration, doping with 50% Ni in the Cr-sites resulted in a half-metallic state with a total moment of nearly zero, a characteristic of spintronic materials. Meanwhile, the optical study reveals that both *ε*_1_^*xx*^ and *ε*_1_^*zz*^ decrease first and then increase rapidly with increasing photon energy up to 1.055 eV. We also found optical anisotropy up to ∼14 eV, where the material becomes almost optically isotropic. This material has a plateau like region in the *σ*_*xx*_ and *σ*_*zz*_ parts of the optical conductivity due to a strong 3d–5d interband transition between Cr and Os. In addition, we performed thermoelectric calculations whose results predict that the material might not be good as a thermoelectric device due to its small power factor.

## Abstract 2

With the help of density functional theory calculations, we explored the recently synthesized double perovskite material Ca_2_CrOsO_6_ and found it to be a ferrimagnetic insulator with a band gap of ∼0.6 eV.

## Graphical abstract

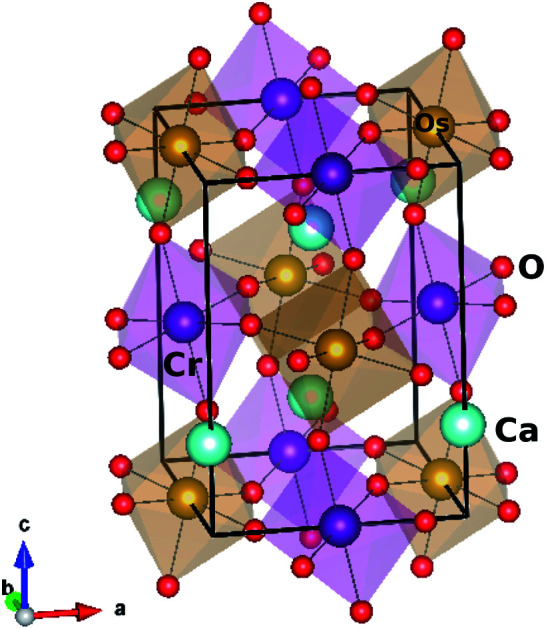

## Introduction

In recent years, research on double perovskites (DPs) with chemical formula A_2_BB′O_6_ (where the B and B′ cations are transition metals and A is an alkaline or rare earth metal) has gained significant interest due to their novel properties arising from the combination of crystal field, spin orbit coupling (SOC) and Coulomb correlation (*U*). Transition metal (TM) DPs are found to show remarkable properties such as structural stability, high charge mobility, finite band gap, superconductivity, half-metallicity (HM), piezoelectricity, thermoelectricity, *etc.*, which can be exploited in modern technological devices.^1–5^ Reports on the realization of room-temperature colossal magnetoresistance and a HM state in Sr_2_FeMoO_6_ and Sr_2_FeReO_6_,^6,7^ multiferroicity in Bi_2_NiMnO_6_,^8^ and magnetodielectricity in La_2_NiMnO_6_ (ref. 9) have motivated researchers to rigorously study these types of materials. In this regard, DPs may help in overcoming today’s major challenges through the development of efficient energy converters and storage devices. TM doped DPs exhibit HM behaviour,^10–13^ in which the material exhibits a conducting state in one of the spin channels while the other spin channel is insulating or semiconducting. HM behaviour has been exhibited by diverse groups of materials such as Heusler alloys, shandites, the Ruddlesden–Popper series, *etc.*,^14–21^ and is a key property for potential applications in spintronics.^22,23^ The majority of experimentally determined HMs are ferromagnets (FMs) such as La_1−*x*_Sr_*x*_MnO_3_, and some are ferrimagnets (FiMs) with finite values of magnetic moments.^6,7,17,18^

Recently, osmium perovskites have been observed to show novel properties. For instance, NaOsO_3_ was found to be magnetically driven by a metal–insulator (MI) transition,^24^ while a ferroelectric type structural transition was observed in LiOsO_3_.^25^ The CsOs_2_O_7_, RbOs_2_O_7_ and KOs_2_O_7_ pyrochlores exhibit unusual superconductivity.^26^ In DPs such as Sr_2_CuOsO_6_ and Sr_2_NiOsO_6_, a MI state was identified,^27^ while HM antiferromagnetism (HMAFM) was predicted in Sr_2_CrOsO_6_ and Sr_2_CrRuO_6_,^1^ and for Ba_2_NiOsO_6_, FM with a Dirac–Mott insulating state was observed near 100 K for the bulk material and anomalous quantum Hall behavior was observed on the surface.^28,29^ In Sr_2_ScOsO_6_, a transition to AFM was observed at 92 K (ref. 30) and a transition to FiM was observed at 725 K.^31^ Similarly, Ca_2_MOsO_6_ (where M = Mn, Fe, Co, and Ni) displays a FiM state with Curie temperature (*T*_C_) of 305, 320, 145, and 175 K, respectively.^32–35^ From density functional theory (DFT) calculations, PrSrMgIrO_6_ was predicted to be an HMAFM with nearly zero effective magnetic moment per unit cell due to the combined effect of Coulomb correlation, *U*, and SOC.^11^ A few DPs such as Ca_2_MgOsO_6_, and Sr_2_MgOsO_6_ have finite gaps at the Fermi level (*E*_F_) due to the correlation effect and are reported to be AFM Mott insulators,^36^ while Sr_2_NiOsO_6_, Sr_2_FeOsO_6_, Sr_2_NiRuO_6_, and SrLaBB′O_6_ (B = Ni, Fe; B′ = Os, Ru)^37,38^ show Mott insulating states under the combined effect of *U* and SOC.

Electron doping has also been considered in FiM DPs such as La_*x*_Ca_2−*x*_CrWO_6_, which demonstrates that doping generally increases the *T*_C_.^39^ DPs are known also as potential candidates for optoelectronic and photovoltaic device applications. The optically active region for these perovskites lies within 3 to 15 eV which is in the range of UV and IR spectra.^40–42^ Frequencies suitable for optoelectronics can be absorbed in this region. Bi_2_FeMnO_6_ is one such example with a band gap of ∼0.8 eV that has been predicted as a suitable material for optical devices.^43^

Recently, Morrow *et al.*^44^ synthesized a new DP material Ca_2_CrOsO_6_ whose *T*_C_ was measured to be much higher than room temperature, *i.e.*, 490 K. This study further reports that the atomic sites occupied by the two transition metals, *i.e.*, Cr and Os cations, are in the ratios of 76% and 24%, respectively, which is a signature of antisite disorder in the system. Here, we are particularly motivated to explore the electronic and related properties of Ca_2_CrOsO_6_ due to (i) its high *T*_C_ above room temperature, (ii) the FiM insulating state, and (iii) the small effective magnetic moment. Upon electron doping, the material is expected to close the band gap in one spin-channel, giving rise to HM state; compensate the total moments nearly to zero; and the increase in *T*_C_.^39^ These features are expected to be important in new DP spintronic devices that work at room temperature.

In this work, we have carried out DFT calculations and found that Ca_2_CrOsO_6_ is a FiM insulator with a total magnetic moment, *μ*_tot_ = 0.23 *μ*_B_ per unit cell. When Ni is partially substituted in the Cr-sites, it contributes an additional five electrons to the system. This significantly changes the electronic as well as magnetic behaviour, giving rise to a nearly compensated HMAFM state. The optical study on Ca_2_CrOsO_6_ shows a strong optical interband transition between the Cr-3d and Os-5d bands in a plateau like region. We further consider the transport properties and calculate the Seebeck coefficient, thermal conductivity and power factor of the parent material Ca_2_CrOsO_6_.

## Crystal structures and computational details

**Fig. 1:**
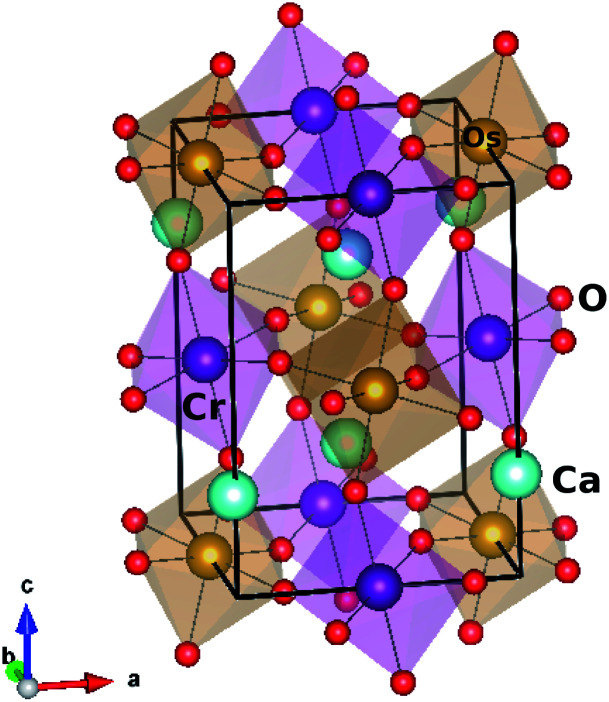
Crystal structure of double perovskite Ca_2_CrOsO_6_.

Ca_2_CrOsO_6_ crystallizes in the monoclinic structure with space group *P*2_1_/*n* (14), and contains CrO_6_ and OsO_6_ octahedra [see Fig. 1]. The experimental lattice parameters chosen for the calculations are *a* = 5.3513 Å, *b* = 5.4561 Å, and *c* = 7.6204 Å, with *β* = 90.092°. The inter-octahedral Cr–O–Os bond angles are 153.3°, 152.6° and 153.8°, with average Cr–O and Os–O bond lengths of 1.972 Å and 1.954 Å respectively.

We performed DFT calculations to explore the electronic, magnetic and related properties of 3D and 2D materials as reported recently.^45–48^ The electronic and magnetic properties were calculated using the full-potential linearized augmented plane wave (FP-LAPW) method as implemented in the WIEN2k code.^49^ The standard generalized gradient approximation (PBE-GGA)^50^ was used, incorporating *U* of 4 eV for Cr (Ni) and 1.5 eV for Os.^35–37^ The SOC was included *via* the second variational step.^51^ The magnetic anisotropy energy (MAE) was calculated by self consistent calculations in full relativistic mode considering five different orientations of the magnetization. The atomic sphere radii *R*_MT_ were 2.14, 1.94, 1.99, 2.01 and 1.64 Bohr for Ca, Cr, Ni, Os and O respectively, and a set of 500 *k*-points within the full Brillouin zone was used which corresponds to an 8 × 8 × 6 *k*-mesh. The convergence was set to 1 mRy per a.u. which gives reliable results for materials that contain transition metals.^52^

In order to consider different magnetic configurations, the symmetry of the crystal has been lowered to *P*1 which corresponds to a total of 20 inequivalent atoms in which three types of oxygen atoms are present and form octahedra with Cr and Os. The tilt and rotation of the CrO_6_ and OsO_6_ octahedra result in Cr–O–Os bond angles within 152.6° to 153.8°. This variation plays a significant role in the magnetic properties.^44^

For the optical property calculations, the full potential local orbital (FPLO) code^53^ (version 18.00) has been used, from which the velocity matrix elements for the optical parameters were extracted. In order to minimize the major fluctuations in the lower energy range, a 16 × 16 × 16 *k*-mesh was used. The electrical transport properties have been calculated using the Boltzmann theory^54^ and the relaxation time approximation as implemented in the Boltztrap code,^55^ interfaced with WIEN2k.^49^ The electrical conductivity and power factor are calculated with respect to time relaxation, *τ*; the Seebeck coefficient is independent of *τ*.

## Results and discussion

### Electronic and magnetic properties

We begin our discussion with the magnetic ground state. For this, we have considered four types of magnetic configurations, one FM state (FM1-↑↑↑↑), two AFM states (AF1-↑↓↑↓, AF2-↑↓↓↑), and one FiM state (FiM-↑↑↓↓). This was done after noting that the nonmagnetic (NM) state of the crystal was found to be the most unstable state, with the highest energy. The FiM configuration is found to be the magnetic ground state with the lowest energy among the other magnetic configurations. The relativistic calculations considered for spin quantisation align along the [100], [010], [001], [110] and [111] directions. For the easy axis along [001], the calculated MAE is 12.75 meV per unit cell.

In the parent material Ca_2_CrOsO_6_, Cr has a charge of +3 with a 3d^3^ configuration, involving the occupancy of the three t_2g_ states in the spin up channel and leaving the e_g_ states empty. As a result, in the density of states (DOS), the three occupied t_2g_ states lie in the valence region while the two empty e_g_ states move to the conduction region. In the spin down channel, all the Cr-d bands are unoccupied and thus lie far into the conduction region. In the case of Os, its charge is +5 with a 5d^3^ configuration. Therefore, only three electrons from Os will occupy the t_2g_ states in the spin down channel, leaving all the other states to shift to the conduction region.

**Fig. 2:**
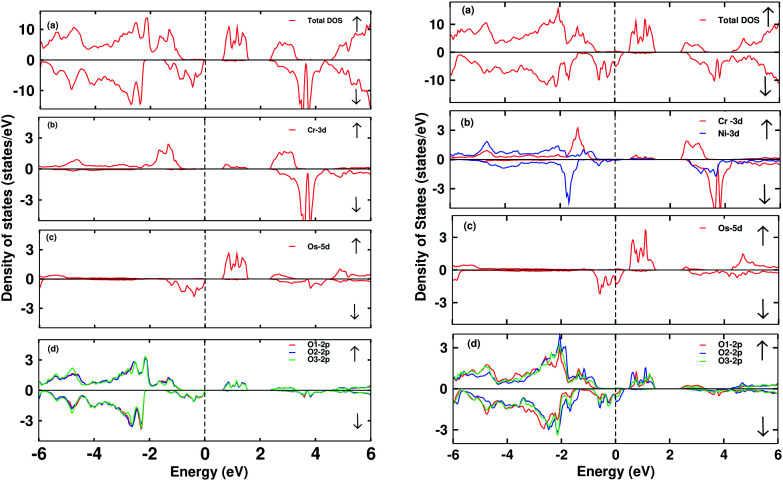
The total and partial density of states (DOS) of Ca_2_CrOsO_6_ (left) and Ca_2_Cr_0.5_Ni_0.5_OsO_6_ (right). (a) Total DOS, (b) partial DOS of Cr-3d and Ni-3d, (c) PDOS of Os-5d, (d) PDOS of O-2p states for spin up (↑) and spin down (↓) channels within the GGA+U+SOC functional. The vertical dotted line indicates *E*_F_ = 0.

**Fig. 3:**
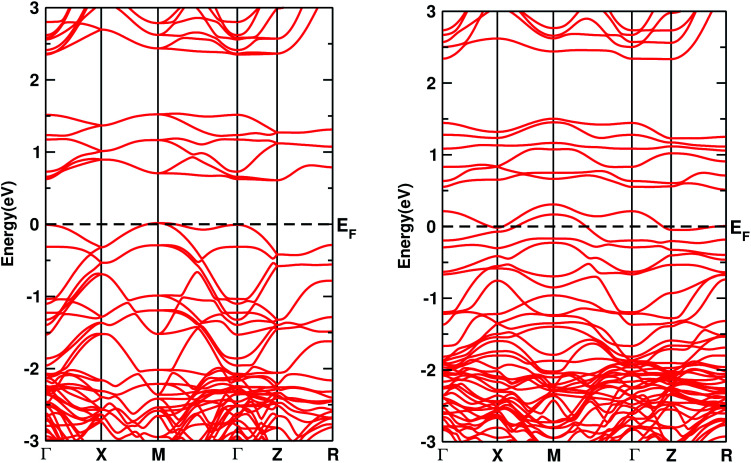
The band structures of Ca_2_CrOsO_6_ (left) and Ca_2_Cr_0.5_Ni_0.5_OsO_6_ (right) within GGA+U+SOC. The horizontal dotted line indicates *E*_F_ = 0.

The electronic DOS and band structures of Ca_2_CrOsO_6_ and Ca_2_Cr_0.5_Ni_0.5_OsO_6_ within GGA+U+SOC are shown in Fig. 2 and 3, respectively. Starting with the parent material Ca_2_CrOsO_6_, a band gap of ∼0.59 eV has been calculated (Fig. 2 and 3 (left)), signifying that Ca_2_CrOsO_6_ is an insulator. The results thus obtained are in good agreement with experimental and theoretical reports.^44,56^ The major contribution to the total DOS around *E*_F_ (Fig. 2(a) (left)) is mainly from the Cr-3d and Os-5d orbitals hybridizing strongly with the O-2p orbitals (Fig. 2(d) (left)).

On the other hand, for the doped material Ca_2_Cr_0.5_Ni_0.5_OsO_6_, one Ni atom is substituted in a Cr-site in Ca_2_CrOsO_6_. This will add five extra electrons to the system as Ni with a charge of +2 has a 3d^8^ configuration. Out of five additional electrons, two occupy the e_g_ states of the spin up channel, while the remaining three are bound to go to the spin down channel to occupy the t_2g_ states. These three electrons of Ni in the spin down channel generate a repulsive effect in the Os-t_2g_ band, which causes the Os band to shift from the valence region towards the conduction region by crossing *E*_F_ in the spin down channel (see Fig. 2(c) (right)). This gives rise to a metallic state in the spin down channel, while the material remains insulating in the spin up channel. Thus, with an insulating state in the spin up channel and a metallic state in the spin down channel, the Ca_2_Cr_0.5_Ni_0.5_OsO_6_ material is predicted to be a HM. It is interesting to note that the HM state remains robust for *U*_Os_ as large as 4 eV. The DOS and band structures for the parent and the doped material within GGA and GGA+U are available in the ESI section for comparison (see Fig. S1–S4†).

**Fig. 4:**
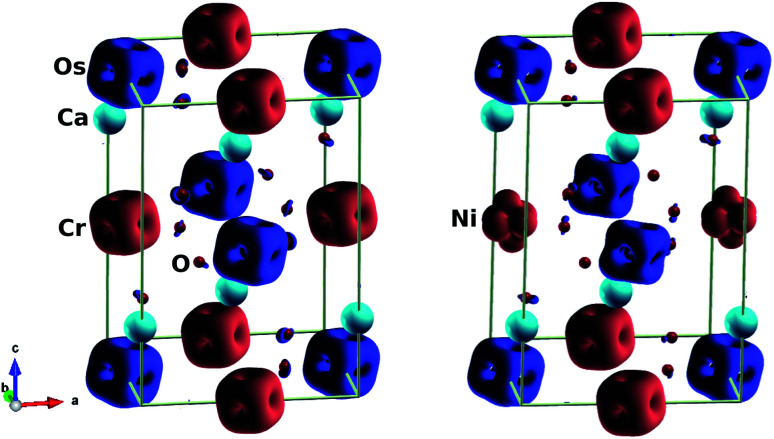
Isosurfaces of spin magnetization density at ±0.21 e Å^−3^ with red (blue) for spin up (down): (left) parent material Ca_2_CrOsO_6_, (right) material with 50% Cr replacement Ca_2_Cr_0.5_Ni_0.5_OsO_6_.

We also considered the magnetic behaviour of Ca_2_Cr_1−*x*_Ni_*x*_OsO_6_. As tabulated in Table 1, for *x* = 0, the individual magnetic moments of the Cr and Os atoms are 2.52 *μ*_B_ and −1.59 *μ*_B_ respectively, with an effective magnetic moment of 0.23 *μ*_B_ per unit cell (2 f.u.). Similarly, for *x* = 0.5, the individual magnetic moments of the Cr, Ni and Os atoms are 2.52 *μ*_B_, 1.64 *μ*_B_ and −1.42 *μ*_B_ respectively, with an effective magnetic moment of 0.21 *μ*_B_ per unit cell. Due to the partial charge transfer from Cr and Os to oxygen, the oxygen atoms are spin polarized in parallel with the Os ions and gain a sizable magnetic moment of −0.07 *μ*_B_, consistent with the isosurface plot shown in Fig. 4 (left). Here, the polarisation is mainly found in the 2p orbitals and this hybridized moment in oxygen increases the resultant magnetic moment.

**Table 1:** Calculated spin magnetic moments (in *μ*_B_) of B (Cr/Ni), B′ (Os) and the 3 types of oxygen atoms, net moments (per unit cell) and band gaps, *E*_g_, (in eV). The calculated orbital moments at the B and B′ sites are shown within parentheses for Ca_2_CrOsO_6_ and Ca_2_Cr_1−*x*_Ni_*x*_OsO_6_

Site	Ca_2_CrOsO_6_				Ca_2_Cr_0.5_Ni_0.5_OsO_6_		
	GGA	GGA+U	GGA+U+SOC	Expt.	GGA	GGA+U	GGA+U+SOC
B	2.20	2.52	2.52(0.05)	2.5	2.19/1.32	2.52/1.65	2.52(0.05)/1.64(0.14)
B′	−1.45	−1.66	−1.59(0.14)	1.26	−1.28	−1.48	−1.42(0.16)
O1	−0.10	−0.11	−0.10		−0.08	−0.09	−0.08
O2	−0.09	−0.10	−0.10		−0.03	−0.05	−0.05
O3	−0.09	−0.10	−0.10		−0.05	−0.07	−0.07
Net	0	0	0.23	0.2	0.0	0	0.21
E_g_	0.53	0.65	0.59		Metallic	Metallic	Metallic

To understand the charge transfer effects among Cr, Ni, Os and O atoms, spin density isosurfaces are drawn as shown in Fig. 4 (right). As observed for the parent compound Ca_2_CrOsO_6_ (see Fig. 4 (left)), the spin density contributions for Cr and Os are due to 3d and 5d states. The observed characteristics are due to the combination of d_*xy*_, d_*xz*_ and d_*yz*_ (*i.e.*, t_2g_) states that are indicative of d^3^ configurations. In addition, the oxygen-2p states become spin-polarized due to the Cr-3d and Os-5d states. When one of the Cr atoms is replaced by Ni, the nature of the spin density changes significantly (see Fig. 4 (right)). Actually, Ni with a charge of +2 has a d^8^ configuration. Thus, with five electrons fully occupying one spin channel, the remaining three electrons move to another spin channel to fill the t_2g_ states. As a result, the remaining visible states are the e_g_ states which are clearly seen in the isosurfaces of the Ni (see Fig. 4 (right)) atoms.

From Table 1, the spin magnetic moment in Os is reduced due to the strong Os–O hybridization, and SOC induces an orbital moment. Thus, the SOC is found to be responsible for the small reduction in spin magnetic moment. The alignment of the orbital magnetic moment of Cr is the same as that of the total spin magnetic moment, showing that the 3d orbital is either semi occupied or more than half filled. Whereas, in the Os-5d state, the spin orientations are antiparallel as compared to the orbital magnetic moments due to the less than half filled states, which is in accordance with Hund’s third rule.^57^ Hence, the orbital magnetic moments on Cr and Os are parallel which increases the net magnetic moment in Ca_2_CrOsO_6_.

### Optical properties

In this work, we have investigated the optical properties of the double perovskite Ca_2_CrOsO_6_, considering the polarization directions *E*_*xx*_ and *E*_*zz*_, as *E*_*xx*_ = *E*_*yy*_ due to the tetragonal symmetry. The optical current *j*_a_ generated by an electric field *E*_b_ is given by: *j*_a_ = *σ*_ab_*E*_b_. For Ca_2_CrOsO_6_, we consider only *E*_*x*_ and *E*_*z*_ polarization as *E*_*x*_ = *E*_*y*_ due to the tetragonal symmetry, leaving only two independent *x* and *z* components of the optical element. The complex dielectric function *ε*(*ω*) = *ε*_1_(*ω*) + i*ε*_2_(*ω*) describes the behavior of materials under incident light, where *ε*_1_(*ω*) and *ε*_2_(*ω*) are the real and imaginary parts of the dielectric function, respectively. The imaginary part *ε*_2_(*ω*) is obtained from the electronic structure through the joint density of states and the momentum matrix elements between the occupied and unoccupied states:1
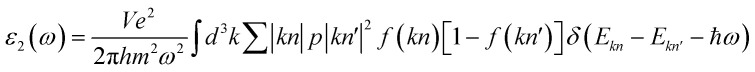
where *p* is the momentum operator, *kn* is the eigenfunction with eigenvalue *E*_*kn*_ and *f*(*kn*) is the Fermi distribution function. The real part of the dielectric function *ε*_1_ is given by the Kramers–Kronig dispersion relations.^58^2
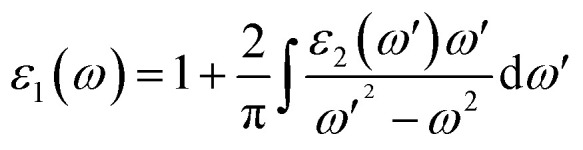


The other optical parameters like optical conductivity *σ*(*ω*) and electron loss function *L*(*ω*) are directly related to the dielectric function as follows.^59^3
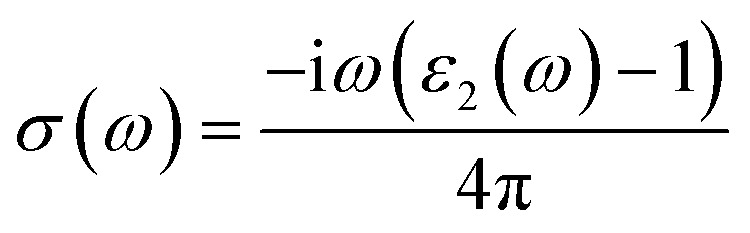
4
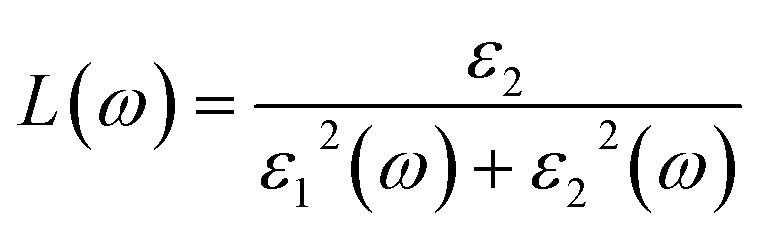


**Fig. 5:**
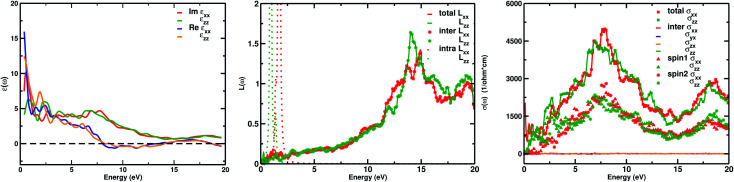
(Left) The real and imaginary parts of the dielectric function *ε*(*ω*) for the interband part. The intraband part of *ε*(*ω*) diverges at *ω* = 0; (middle) total, intraband and interband loss function *L*(*ω*); (right) total, interband and individual spin contributions to optical conductivity *σ*(*ω*). Here, for Ca_2_CrOsO_6_, *σ*_*xx*_(*ω*) and *σ*_*zz*_(*ω*) contribute.

The real part of the complex dielectric function *ε*_1_(*ω*) expresses the electronic polarizability of materials due to incident photons. Fig. 5 (left) shows the variation of the dielectric function (real and imaginary) as a function of photon energy. The calculated static dielectric constants for *x* and *z* polarization (*ε*_1_^*xx*^(0) and *ε*_1_^*zz*^(0)) are 15.94 eV and 12.43 eV, respectively (Table 2). Both *ε*_1_^*xx*^ and *ε*_1_^*zz*^ decrease first and then increase rapidly with increasing photon energy up to 1.055 eV. With a small increase in photon energy above 0.0 eV, *ε*_1_^*zz*^ decreases more as compared to *ε*_1_^*xx*^ and then increases again. At a certain energy, both *ε*_1_^*xx*^ and *ε*_1_^*zz*^ show a maximum peak where *ε*_1_^*xx*^ surpasses *ε*_1_^*zz*^ in magnitude. The plot also indicates the presence of optical anisotropy up to ∼14 eV, and after that the material becomes almost optically isotropic. Both *ε*_1_^*xx*^ and *ε*_1_^*zz*^ become zero at ∼7.6 eV which may be due to the occurrence of plasmonic type oscillations. We know that the imaginary part *ε*_2_(*ω*) of the complex dielectric function is related to optical absorption. The dependence of *ε*_2_(*ω*) on photon energy is presented in Fig. 5 (left).

**Table 2:** Calculated optical parameters at the main peak value for the Ca_2_CrOsO_6_ compound

Optical parameters	*ε*_1_^*xx*^(0)	*ε*_1_^*zz*^(0)	*L*(*ω*)	*σ*_1_^*xx*^(*ω*)	*σ*_1_^*zz*^(*ω*)
eV	15.94	12.43	14.11	7.79	7.32

From the figure, we notice a rapid increase in *ε*_2_(*ω*) beyond the threshold energy which corresponds to the calculated energy band gap and observed a maximum peak at 1.0–1.5 eV. After that, the value of *ε*_2_(*ω*) decreases with severe fluctuations. A significant plateau like region is obtained in the energy range from 3.0 eV up to 10.0 eV. This can also be verified by the optical conduction plot which shows active absorption of photon energy due to direct electron transitions. The variation of the electron energy loss function *L*(*ω*) *versus* photon energy is also plotted in Fig. 5 (middle). *L*(*ω*) gives a description of the energy loss due to the scattering of a fast electron travelling in the material. The electron energy loss is very high in the low energy region in the case of intraband transitions. However, for interband transitions, *L*(*ω*) slowly increases up to 12.0 eV and then abruptly increases beyond 13.0 to 14.0 eV. The low value of electron energy loss is due to the fact that *L*(*ω*) is inversely related to *ε*_2_(*ω*). The large peak in the energy loss spectra at around 13.0 eV to 14.0 eV corresponds to a plasmon frequency *ω*_p_ at 13.0–14.0 eV. We also calculated the optical conductivity *σ*(*ω*) as shown in Fig. 5 (right). The only independent components of *σ*(*ω*) in this material are *σ*_*xx*_(*ω*), *σ*_*zz*_(*ω*), *σ*_*yx*_(*ω*) and *σ*_*zx*_(*ω*). *σ*_*yx*_(*ω*) and *σ*_*zx*_(*ω*) are zero, and only the *σ*_*xx*_(*ω*) and *σ*_*zz*_(*ω*) components contribute. The optical conductivity of Ca_2_CrOsO_6_ has a plateau like region at 3–12 eV rather than a sharp peak like feature. This indicates a strong optical interband transition between the Cr-3d and Os-5d bands in this region.

### Thermoelectric properties

The insulating behavior of Ca_2_CrOsO_6_ prompted us to investigate the thermoelectric properties. The performance of thermoelectric materials is given by a dimensionless parameter called the figure of merit, *i.e.*, *ZT* = *S*^2^*σT*/*κ*, where *S*, *σ* and *κ* represent the Seebeck coefficient, electrical conductivity, and thermal conductivity, respectively.^60–62^ Conventional materials such as Bi_2_Te_3_ and PbTe have shown a promising figure of merit, however, these alloys suffer from toxic compositions, low thermal stability, and expensive constituents. In this regard, oxide materials could be a promising alternative owing to their environmental friendliness, non-toxicity, good thermal stability, and low-cost compositions.^63,64^ Recently, good thermoelectric performances have been reported for double perovskite oxides.^65,66^ Motivated by these findings, we investigated the thermoelectric properties of Ca_2_CrOsO_6_ utilizing the Boltztrap code which is used for computing the electrical transport coefficients *S* and *σ*, and thereby *S*^2^*σ*, within the rigid band approximation (RBA) and constant relaxation time approach (CRTA). The CRTA assumes the Seebeck coefficient to be independent of relaxation time, *τ*, and the electrical conductivity and power factor (PF) are expressed with respect to *τ*. These two approaches have been successful in the theoretical investigation of new thermoelectric materials by many groups.^67–70^

**Fig. 6:**
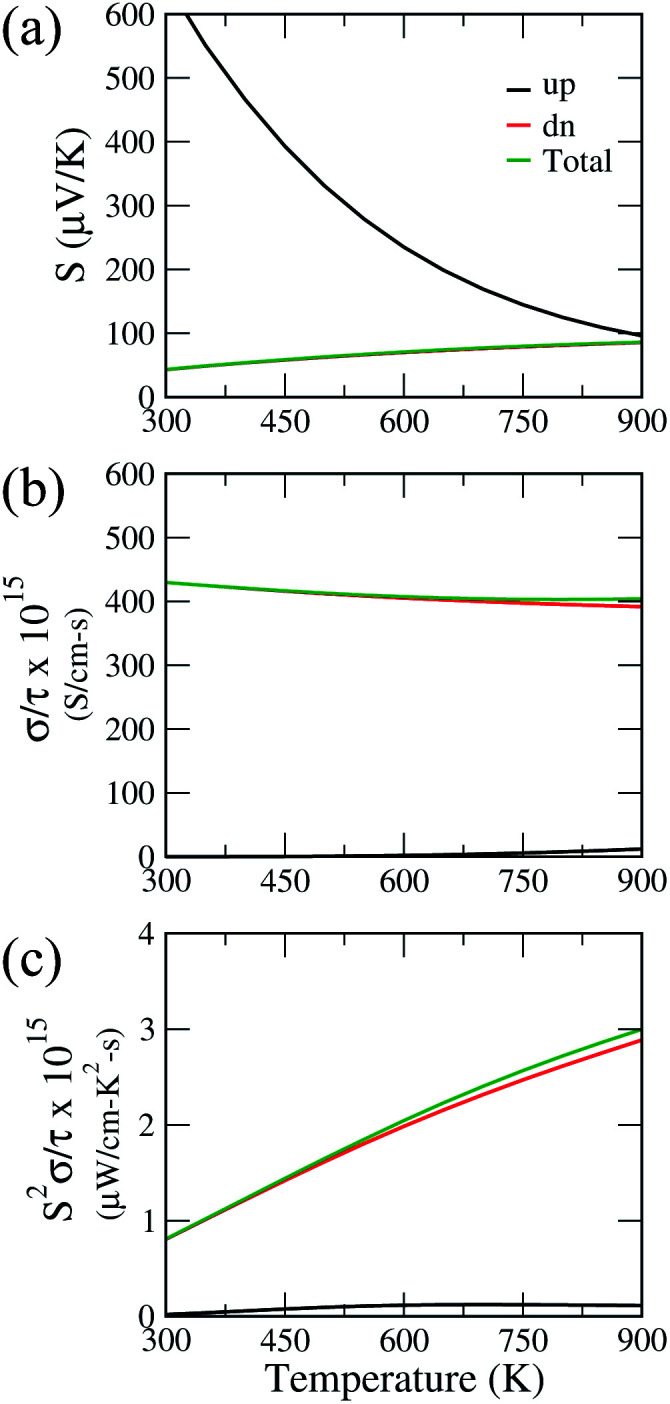
(a) Seebeck coefficient, (b) electrical conductivity, and (c) power factor of Ca_2_CrOsO_6_ as a function of temperature for spin up (black), spin down (red) and total contribution (green). The electrical conductivity and power factor are plotted with respect to the relaxation time.

Fig. 6 shows the Seebeck coefficient, electrical conductivity and power factor with respect to relaxation time, as a function of temperature for Ca_2_CrOsO_6_ for the spin up and spin down channels, and the total contribution. The parameters are calculated for both the spin channels according to the two current model.^71^ Within this model, the total electrical conductivity and Seebeck coefficients are given by *σ* = *σ*(↑) + *σ*(↓) and *S* = [*S*(↑)*σ*(↑) + *S*(↓)*σ*(↓)]/[*σ*(↑) + *σ*(↓)] where (↑) and (↓) represent the coefficients for spin up and spin down respectively. Utilizing this model, the total Seebeck coefficient obtained shows an increasing trend in the temperature range 300–900 K. The positive values are in the range 43–83 μV K^−1^ and are indicative of p-type charge carriers. However, the Seebeck coefficient values are not significant as compared to benchmark values (150 μV K^−1^).^72^ For evaluating the electrical conductivity and power factor, one needs to incorporate the relaxation time which is not trivial to calculate. The relaxation time is very sensitive as it may vary for different systems at different doping levels and temperatures.^73^ Thus, the actual value of the relaxation time is quite essential for determining the transport properties. However, in previous theoretical studies, a relaxation time of 5 × 10^−15^ s has been used for evaluating the transport properties.^74^ Utilizing the same value, we obtained good values of total electrical conductivity in the range 429–404 S cm^−1^ in the temperature range 300–900 K. These values can be attributed to the greater density of states at the VBM owing to the degenerate bands. The degenerate bands increase the density of states which results in a larger number of charge carriers. The greater density of states at the VBM close to the Fermi level as compared to the conduction band minimum suggests that hole doping would be beneficial for improving the transport properties.^75^

The calculated total power factor with respect to the relaxation time is not significant, owing to the low Seebeck coefficient. The values range from 0.08 μW cm^−1^ K^−2^ s^−1^ at room temperature to 3 μW cm^−1^ K^−2^ s^−1^. Utilizing the relaxation time as before, *i.e.*, *τ* = 5 × 10^−15^ s, the maximum obtained power factor is 15 μW cm^−1^ K^−2^ at 900 K. The proposed value, though not significant, can be further improved by utilizing suitable dopants and synthesis approaches, meriting further experimental investigations.

## Conclusions

By means of density functional calculations, we found that the double perovskite material Ca_2_CrOsO_6_ is a ferrimagnetic insulator. Upon substantial doping with Ni (50%) in the Cr-sites, the composition Ca_2_Cr_0.5_Ni_0.5_OsO_6_ shows nearly compensated half-metal behaviour which is desirable for spintronic device applications. The optical study of the parent material Ca_2_CrOsO_6_ reveals that both *ε*_1_^*xx*^ and *ε*_1_^*zz*^ decrease first and then increase rapidly with increasing photon energy up to 1.055 eV. At a certain energy, both *ε*_1_^*xx*^ and *ε*_1_^*zz*^ show a maximum peak where *ε*_1_^*xx*^ surpasses *ε*_1_^*zz*^ in magnitude. We also found that optical anisotropy is present up to ∼14 eV. The promising optical response of Ca_2_CrOsO_6_ due to Cr-3d and Os-5d interband transitions makes it an important material for photovoltaic and optoelectronic applications. In addition, we investigated the thermoelectric properties, however, the material does not show promise as a thermoelectric device.

## Conflicts of interest

There are no conflicts to declare.

## Supplementary Material

RA-010-C9RA10775D-s001

## References

[cit1] Lee K. W., Pickett W. E. (2008). Phys. Rev. B: Condens. Matter Mater. Phys..

[cit2] Liu Y. P., Fuh H. R., Wang Y. K. (2014). Comput. Mater. Sci..

[cit3] Reshak A. H. (2016). Phys. Chem. Chem. Phys..

[cit4] Rai D. P., Shankar A., Ghimire M. P., Thapa R. K. (2015). Comput. Mater. Sci..

[cit5] Anderson M. T., Greenwood K. B., Taylor G. A., Poeppelmeier K. R. (1993). Prog. Solid State Chem..

[cit6] Tokura Y. (2006). Rep. Prog. Phys..

[cit7] Kobayashi K.-I., Kimura T., Sawada H., Terakura K., Tokura Y. (1998). Nature.

[cit8] Shimakawa Y., Azuma M., Ichikawa N. (2011). Materials.

[cit9] Rogado N. S., Li J., Sleight A. W., Subrama- nian M. A. (2005). Adv. Mater..

[cit10] Pickett W. E. (1998). Phys. Rev. B: Condens. Matter Mater. Phys..

[cit11] Ghimire M. P., Wu L. H., Hu X. (2016). Phys. Rev. B.

[cit12] Hu X. (2012). Adv. Mater..

[cit13] Nie Y.-M., Hu X. (2008). Phys. Rev. Lett..

[cit14] Sargolzaei M., Richter M., Koepernik K., Opahle I., Eschrig H. (2006). Phys. Rev. B: Condens. Matter Mater. Phys..

[cit15] Ghimire M. P., Facio J. I., You J.-S., Ye L., Checkelsky J. G., Fang S., Kaxiras E., Richter M., van den Brink J. (2019). Phys. Rev. Res..

[cit16] Pickett W. E., Moodera J. S. (2001). Phys. Today.

[cit17] Muller G. M., Walowski J., Djordjevic M., Miao G.-X., Gupta A., Ramos A. V., Gehrke K., Moshnyaga V., Samwer K., Schmalhorst J., Thomas A., Hütten A., Reiss G., Moodera J. S., Münzenberg M. (2009). Nat. Mater..

[cit18] Shibata G., Kitamura M., Minohara M. (2018). et al.. npj Quantum Mater..

[cit19] Ruddlesden S. N., Popper P. (1957). Acta Crystallogr..

[cit20] Ghimire M. P., Thapa R. K., Rai D. P., Sandeep, Sinha T. P., Hu X. (2015). J. Appl. Phys..

[cit21] van Leuken H., de Groot R. A. (1995). Phys. Rev. Lett..

[cit22] Felser C., Fecher G. H., Balke B. (2007). Angew. Chem., Int. Ed..

[cit23] Katsnelson M. I., Irkhin Y. Yu., Chioncel L., Licht-enstein A. I., de Groot R. A. (2008). Rev. Mod. Phys..

[cit24] Calder S., Garlea V. O., McMorrow D. F., Lumsden M. D., Stone M. B., Lang J. C., Kim J. W., Schlueter J. A., Shi Y. G., Yamaura K., Sun Y. S., Tsujimoto Y., Christianson A. D. (2012). Phys. Rev. Lett..

[cit25] Shi Y. G., Guo Y. F., Wang X., Princep A. J., Khalyavin D., Manuel P., Michiue Y., Sato A., Tsuda T., Yu S., Arai M., Shirako Y., Akaogi M., Wang N. L., Yamaura K., Boothroyd A. T. (2013). Nat. Mater..

[cit26] Hiroi Z., Yamaura J., Hattori K. (2012). J. Phys. Soc. Jpn..

[cit27] Tian A. C., Wibowo H. C., Loye Z., Whangbo M. H. (2011). Inorg. Chem..

[cit28] Feng H. L., Calder S., Ghimire M. P., Yahua Y., Shirako Y., Tsujimoto Y., Matsushita Y., Hu Z., Kuo C.-Y., Tjeng L. H., Pi T.-W., Soo Y.-L., He J., Tanaka M., Katsuya Y., Richter M., Yamaura K. (2016). Phys. Rev. B.

[cit29] Lu H.-S., Guo G.-Y. (2019). Phys. Rev. B.

[cit30] Taylor A. E., Morrow R., Singh D. J., Calder S., Lumsden M. D., Woodward P. M., Christianson A. D. (2015). Phys. Rev. B: Condens. Matter Mater. Phys..

[cit31] Krockenberger Y., Mogare K., Reehuis M., Tovar M., Jansen M., Vaitheeswaran G., Kanchana V., Bultmark F., Delin A., Wilhelm F., Rogalev A., Winkler A., Alff L. (2007). Phys. Rev. B: Condens. Matter Mater. Phys..

[cit32] Feng H. L., Arai M., Matsushita Y., Tsujimoto Y., Guo Y., Sathish C. I., Wang X., Yuan Y. H., Tanaka M., Yamaura K. (2014). J. Am. Chem. Soc..

[cit33] Ghimire M. P., Hu X. (2016). Mater. Res. Express.

[cit34] Morrow R., Samanta K., Saha-Dasgupta T., Xiong J., Freeland J. W., Haskel D., Woodward P. M. (2016). Chem. Mater..

[cit35] Feng H. L., Ghimire M. P., Hu Z., Liao S.-C., Agrestini S., Chen J., Yuan Y., Matsushita Y., Tsujimoto Y., Katsuya Y., Tanaka M., Lin H.-J., Chen C.-T., Weng S.-C., Valvidares M., Chen K., Baudelet F., Tanaka A., Greenblatt M., Tjeng L. H., Yamaura K. (2019). Phys. Rev. Mater..

[cit36] Yuan Y., Feng H. L., Ghimire M. P., Matsushita Y., Tsujimoto Y., He J., Tanaka M., Katsuya Y., Yamaura K. (2015). Inorg. Chem..

[cit37] Yadav D. K., Bhandari S. R., Belbase B. P., Kaphle G. C., Rai D. P., Ghimire M. P. (2019). Comput. Mater. Sci..

[cit38] Song W., Zhao E., Meng J., Wu Z. (2009). J. Chem. Phys..

[cit39] Geprags S., Majewski P., Gross R. (2006). J. Appl. Phys..

[cit40] Nabi M., Gupta D. C. (2019). RSC Adv..

[cit41] Parrey K. A., Khandy S. A., Islam I., Laref A., Gupta D. C., Niazi A., Aziz A., Ansari S. G., Khenata R., Rubab S. (2018). J. Electron. Mater..

[cit42] Roknuzzaman M., Zhang C., Ostrikov K., Du A., Wang H., Wang L., Tesfamichael T. (2019). Sci. Rep..

[cit43] Ahmed T., Chen A., Yarotski D. A., Trugman S. A., Jia Q., Zhu J.-X. (2017). APL Mater..

[cit44] Morrow R., Soliz J. R., Hauser A. J., Gallagher J. C., Susner M. A., Sumption M. D., Aczel A. A., Yan J., Yang F., Woodward P. M. (2016). J. Solid State Chem..

[cit45] Bhandari S. R., Thapa R. K., Ghimire M. P. (2015). J. Nep. Phys. Soc..

[cit46] Rafique M., Yong S., Tan H. (2017). Phys. E..

[cit47] Rafique M., Unar M. A., Ahmed I., Chachar A. R., Shuai Y. (2018). J. Phys. Chem. Solids.

[cit48] Rafique M., Yong S., Tan H. (2017). J. Mater. Chem. C.

[cit49] Blaha P. , Schwarz K., Madsen G. K. H., Kvasnicka D. and Luitz J., An Augmented Plane Wave Plus Local Orbitals Program for Calculating Crystal Properties, Technische Universität Wien, Vienna, Austria, 2001, ISBN 3-9501031-1-2

[cit50] Perdew J. P., Burke K., Ernzerhof M. (1996). Phys. Rev. Lett..

[cit51] Kunes J., Novak P., Divis M., Oppeneer P. M. (2001). Phys. Rev. B: Condens. Matter Mater. Phys..

[cit52] Muhammad R., Uqaili M. A., Shuai Y., Mahar M. A., Ahmed I. (2018). Appl. Surf. Sci..

[cit53] Koepernik K., Eschrig H. (1999). Phys. Rev. B: Condens. Matter Mater. Phys..

[cit54] Allen P. B. , Boltzmann theory and resistivity of metals in Quantum Theory of Real Materials, ed. J. R. Chelikowsky and S. G. Louie, Kluwer, Boston, 1996

[cit55] Madsen G. K. H., Singh D. J. (2006). Comput. Phys. Commun..

[cit56] Zu N., Li R., Ai R. (2018). J. Magn. Magn. Mater..

[cit57] Jeng H. T., Guo G. Y. (2003). Phys. Rev. B: Condens. Matter Mater. Phys..

[cit58] Toll J. S. (1956). Phys. Rev..

[cit59] Hoat D., Silva J. R., Blas A. M. (2019). J. Solid State Chem..

[cit60] Sevincli H., Sevik C., Cagin T., Cuniberti G. (2013). Sci. Rep..

[cit61] Boona S. R. (2017). Nature.

[cit62] Tan G., Hao S., Zhao J., Wolverton C., Kanatzidis M. G. (2017). J. Am. Chem. Soc..

[cit63] Roy P., Waghmare V., Maiti T. (2016). RSC Adv..

[cit64] Saxena M., Tanwar K., Maiti T. (2017). Scr. Mater..

[cit65] Roy P., Bose I., Maiti T. (2016). Integr. Ferroelectr..

[cit66] Sugahara T., Ohtaki M., Souma T. (2008). J. Ceram. Soc. Jpn..

[cit67] Madsen G. K. H. (2006). J. Am. Chem. Soc..

[cit68] Yang J., Li H., Wu T., Zhang W., Chen L., Yang J. (2008). Adv. Funct. Mater..

[cit69] Lee M.-S., Poudeu F. P., Mahanti S. D. (2011). Phys. Rev. B: Condens. Matter Mater. Phys..

[cit70] Zeeshan M., Singh H. K., van den Brink J., Kandpal H. C. (2017). Phys. Rev. Mater..

[cit71] Sharma S., Panday S. K. (2015). Phys. Lett. A.

[cit72] Tritt T. M. (2011). Annu. Rev. Mater. Res..

[cit73] Zeeshan M., van den Brink J., Kandpal H. C. (2017). Phys. Rev. Mater..

[cit74] Parrey K. A., Khandy S. A., Islam I., Laref A., Gupta D. C., Niazi A., Aziz A., Ansari S. G., Khenata R., Rubab S. (2018). J. Electron. Mater..

[cit75] Zeeshan M., Nautiyal T., van den Brink J., Kandpal H. C. (2018). Phys. Rev. Mater..

