# Supplementary material for: Electronic, magnetic, optical and thermoelectric properties of Ca2Cr1−xNixOsO6 double perovskites
Source: RSC Adv. 2020 Apr 23;10(27):16179–86. doi: 10.1039/c9ra10775d (PMC9059137; doi:10.1039/c9ra10775d)
Supplement: RA-010-C9RA10775D-s001 [file RA-010-C9RA10775D-s001.pdf]

Electronic Supplementary Material (ESI) for RSC Advances.
This journal is © The Royal Society of Chemistry 2020

Journal Name

ARTICLE TYPE

Cite this: DOI: 10.1039/xxxxxxxxxx

Supplementary: Electronic, Magnetic, Optical and Thermoelectric Properties of $Ca_2Cr_{1-x}Ni_xOsO_6$ Double Perovskites

Shalika R. Bhandari,[a,b,c] D. K. Yadav,[c] B. P. Belbase,[a,c] M. Zeeshan,[d] B. Sadhukhan,[b] D. P. Rai,[e], R. K. Thapa,[c], G. C. Kaphle,[a,c] and Madhav Prasad Ghimire[a,b,c*]

Received Date
Accepted Date

DOI: 10.1039/xxxxxxxxxx

www.rsc.org/journalname

With the help of density functional theory calculations, we explored a recently synthesized double perovskite material Ca_2CrOsO_6 and found it to be ferrimagnetic insulator with a band gap of ∼0.6 eV. The effective magnetic moment of it is found to be ∼0.23 μ_B/unitcell. The proposed behavior arises from the cooperative effect of spin-orbit coupling and Coulomb correlation of Cr-3d and Os-5d electrons along with the crystal field. Within the ferrimagnetic configuration, doping of 50% by Ni to the Cr-site resulted in the half-metallic state with compensation of total moments nearly to zero, a characteristic of spintronic materials. Meanwhile, the optical study reveals that both ε_1^{xx} and ε_1^{zz} decreases first and increases rapidly with increasing photon energy up to 1.055 eV. We also found the optical anisotropy up to ∼14 eV, which becomes almost optically isotropic. This material has a plateau like region in the σ_{xx} and σ_{zz} part of the optical conductivity due to a strong $3d$-$5d$ interband transition between Cr and Os. In addition, we performed the thermoelectric calculations whose results predicts that the material might not be good as a thermoelectric device due to small power factor.

[a] *Central Department of Physics, Tribhuvan University, Kathmandu, Nepal*
[b] *Institute for Theoretical Solid State Physics, IFW Dresden e. V., Dresden-01069, Germany*
[c] *Condensed Matter Physics Research Center (CMPRC), Butwal, Rupandehi, Nepal*
[d] *Department of Chemistry, Indian Institute of Technology-Roorkee, Roorkee 247667, Uttarakhand, India*
[e] *Physical Sciences Research Center (PSRC), Department of Physics, Pachhunga University College, Aizawl, Mizoram, India*
[*] *Corresponding author E-mail: mpghimire@tucdp.edu.np; ghimire.mpg@gmail.com*

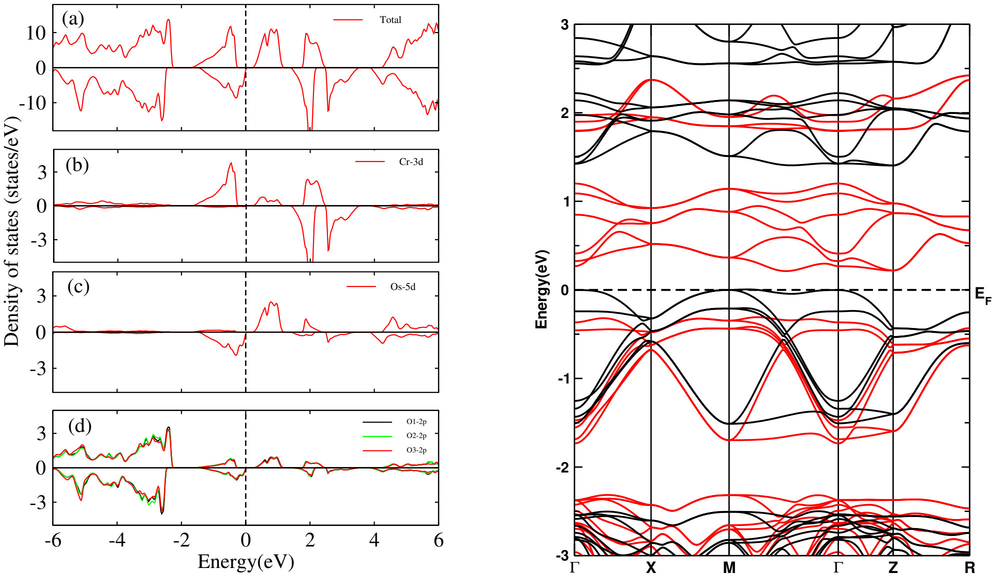

Fig. S1 (Color online) DOS (top) and band structure (bottom) of Ca_2CrOsO_6 with GGA functional. The vertical in (up) and horizontal in (down) dotted line indicates E_F=0.

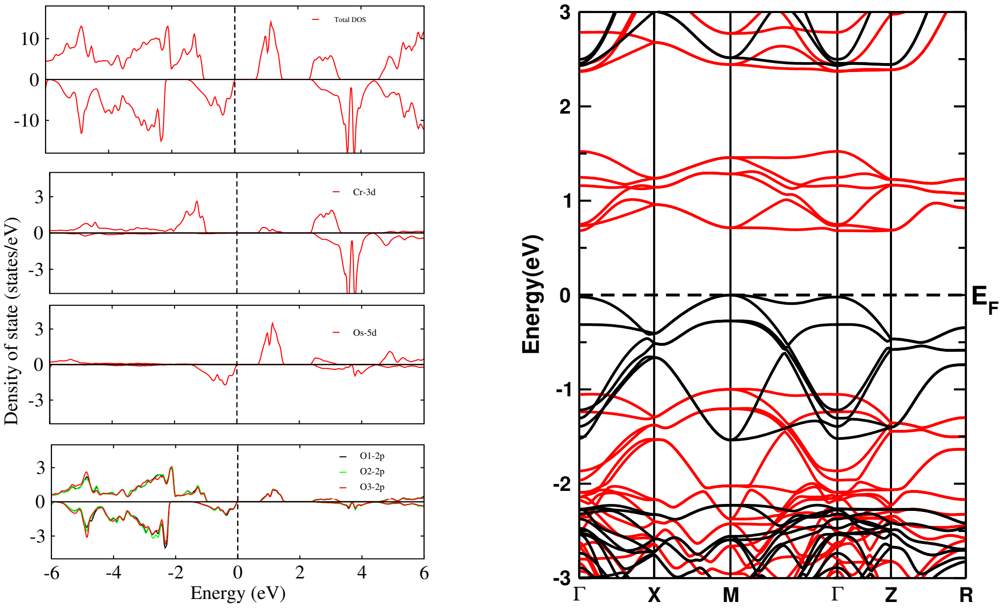

Fig. S2 (Color online) DOS (left) and band structure (right) of Ca_2CrOsO_6 with GGA+U functional. The vertical in (left) and horizontal in (right) dotted line indicates E_F=0.

This journal is © The Royal Society of Chemistry [year]

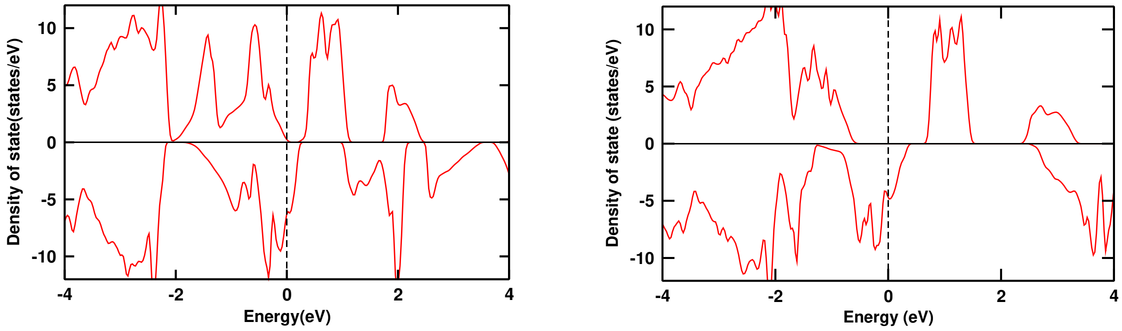

Fig. S3 (Color online) Total DOS of $Ca_2Cr_{0.5}Ni_{0.5}OsO_6$ with GGA (left) and GGA+U (right) functional. The vertical dotted line indicates E_F=0.

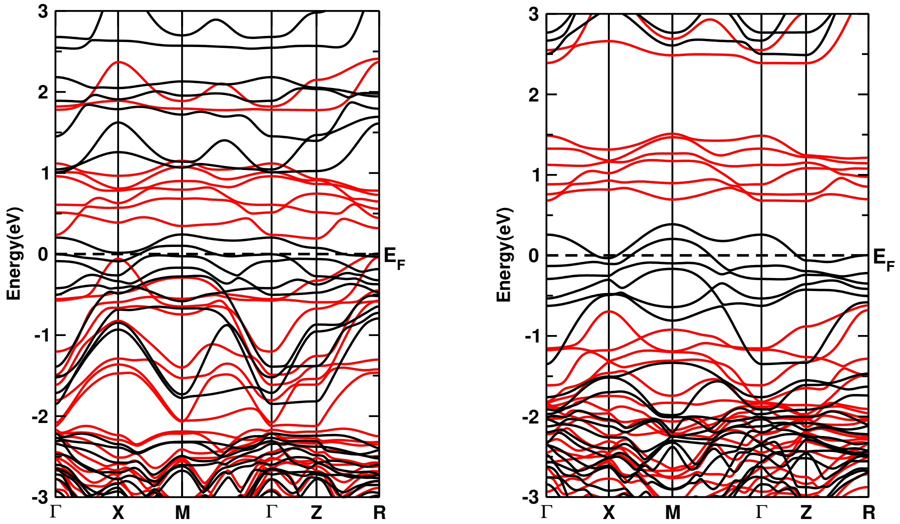

Fig. S4 (Color online) Band structure of $Ca_2Cr_{0.5}Ni_{0.5}OsO_6$ with GGA (left) and GGA+U (right) functional. The horizontal dotted line indicates E_F=0.
